# Patient centered outcomes in stroke: utility-weighted modified Rankin Scale results in a community-based study

**DOI:** 10.3389/fneur.2025.1539107

**Published:** 2025-03-21

**Authors:** Carlos Delfino, Gabriel Cavada, Lorena Hoffmeister, Pablo Lavados, Paula Muñoz Venturelli

**Affiliations:** ^1^Instituto de Ciencias e Innovación en Medicina, Facultad de Medicina Clínica Alemana Universidad del Desarrollo, Santiago, Chile; ^2^Unidad de Investigación y Ensayos Clínicos, Clínica Alemana de Santiago, Facultad de Medicina Clínica Alemana Universidad del Desarrollo, Santiago, Chile; ^3^Servicio de Neurología, Departamento de Neurología y Psiquiatría, Clínica Alemana de Santiago, Facultad de Medicina Clínica Alemana Universidad del Desarrollo, Santiago, Chile; ^4^Faculty of Medicine, The George Institute for Global Health, University of New South Wales, Sydney, NSW, Australia

**Keywords:** patient-centered outcomes, stroke, utility-weighted, modified Rankin Scale, community-based study

## Abstract

**Background and aims:**

The transformation of modified Rankin Scale (mRS) scores based on the corresponding utilities of health-related quality of life questionnaires can facilitate the capture of Patient-Centered Outcomes (PCO) in stroke. We aimed to derive utility-weighted modified Rankin Scale (UW-mRS) values by mapping mRS functional status to EQ-5D-3L scores in a population-based cohort of stroke patients.

**Methods:**

The UW-mRS was obtained by analyzing the EQ5-D-3 L and mRS scores at 180 days after any stroke in the ÑANDU study, a large prospective community-based study in Chile. The mRS prediction was estimated using a linear regression adjusted by the EQ-5D-3L value. Generalized linear and binary logistic regression models were constructed to determine influencing factors of the UW-mRS, using STATA software (version 18.0).

**Results:**

We included 773 patients presenting with any stroke during 2015–2016: 48% were female, with a mean age of 71 years (SD 13.8), and 85% had an acute ischemic stroke (AIS). 82% of patients had a low socioeconomic status, 50% had less than 12 years of formal education, and only 32% lived in urban areas. UW-mRS values for mRS categories 0–6 at 180 days were 0.913, 0.694, 0.425, 0.249, −0.102, −0.347 and 0, respectively. Multivariable analysis identified age > 70 years (Coefficient *β* [β] -0.038 [Standard error SE 0.018], *p* = 0.032), prior mRS score 3–5 (*β* −0.556 [SE 0.197], *p* < 0.001), ischemic stroke (β −0.066 [SE 0.025], *p* = 0.010), and National Institutes of Health Stroke Scale (NIHSS) at admission>5 (*β* −0.015 [SE 0.002], *p* < 0.001) as significant predictors of worse UW-mRS scores (R^2^ = 70%) in the overall group. Sex-disaggregated analysis showed that age > 70 years was a significant predictor in males (β −0.069 [SE 0.024], *p* = 0.006), while presenting an AIS had a greater impact on female’s worse UW-mRS score (β −0.087 [SE 0.033], *p* = 0.010).

**Conclusion:**

These results present UW-mRS values derived from a population-based stroke study. Key determinants of health-related quality of life in post-stroke patients included age, prior disability, and stroke severity. Sex-disaggregated analysis revealed age being significant for males and AIS for females. Incorporating PCO as UW-mRS in stroke research can provide a more nuanced understanding of the impact of stroke on survivors, offering valuable insights for clinical decision-making and rehabilitation strategies across diverse healthcare contexts.

## Introduction

According to the most recent Global Burden of Disease (GBD) study, stroke remains one of the leading cause of death and disability combined worldwide (1). Between 1990 to 2019, the global burden of stroke, in terms of absolute number of cases, increased substantially, with the majority (86.0% of deaths and 89.0% of disability-adjusted life years, DALYs) residing in low- and lower-middle-income countries (LMICs) (2). Given the wide range of functional disability among stroke survivors, it is crucial to accurately measure and classify these impairments.

Several scales have been developed to categorize stroke patients. The modified Rankin Scale (mRS), a seven-level scale of global impairment and disability is widely used as a functional outcome measure in both clinical research and practice (3). While the mRS provides valuable insights into functional status, it does not reflect the broader impact on quality of life (4). Moreover, its power is limited when analyzed dichotomously and its indication of effect size is difficult to interpret when analyzed ordinally (5). Therefore, the development of a utility-weighted modified Rankin scale (UW-mRS), which incorporates a health utility scale as Patient Centered Outcome (PCO), is recommended and has been used in several recent clinical trials (6, 7).

Health utility weights represent the preference for a specific health outcome, allowing comparison of quality of life across different clinical settings (8). They range from perfect health (a score of 1) to outcomes worse than death (where death is scored as 0 and negative values indicate worse-than-death states). The utility approach offers several advantages: it aligns with the principles of economic evaluation, enables broad comparisons, and provides a detailed view of patients’ experiences, highlighting both improvements and declines in health status (8). Despite these benefits, the application of UW-mRS outside the clinical setting remains limited (9).

The aim of this study was to incorporate the quality-of-life perspective into functional scales and analyze its determinants, by developing the UW-mRS as an outcome measure for patients 180 days after suffering a stroke, using data from the Ñuble population between 2015 and 2017.

## Materials and methods

Individual participant data were pooled from the ÑANDU study, a large prospective community-based study in Chile, whose methodology and results have been previously published (10). At 180 days after the event, trained personnel conducted telephone interviews to evaluate the patients. Information was collected on recovery, dependency, and health-related quality of life.

### Instruments

The mRS is a widely used tool for assessing health outcomes in stroke patients (11). The mRS evaluates the level of disability by considering activity limitations and lifestyle changes. The scale has 7 grades, from 0 to 6: 0 means no symptoms, 5 means severe disability, and 6 indicates death (3).

The EuroQol EQ-5D-3L is a questionnaire designed to measure a patient’s health status preferences (12). It consists of 5 dimensions: mobility, self-care, usual activities, pain, and anxiety. Each dimension has 3 levels: no problems, some problems, and extreme problems, coded from 1 to 3 (13). The EQ-5D-3L health states are represented by a sequence of 5 numbers that describe each level within each dimension. For example, 11111 indicates perfect health, while 33333 represents the worst possible health state. The system defines 243 possible health states, each of which can be supplemented using a scoring or weighting system to convert profile data into a single numerical value: the EQ-5D-3L values (14). These scoring systems are typically preference-based, meaning that the problems in each dimension are weighted to reflect public perception of their severity. The EQ-5D-3L index values are constructed on a scale anchored at 1, representing full health, and 0, representing death (14).

The EQ-5D-3L value set was selected from a previous study conducted in Chile, which evaluated the health status of the general population using the Time Trade-Off technique (15). Patients who died during follow-up (mRS = 6) were assigned an EQ-5D-3L value of 0 (zero).

## Statistical analyses

Quantitative variables were reported as means (SD) or medians (IQR) depending on normality (using K-S test) and were compared according normal/ non-normal distribution using the T test or Mann–Whitney U test. Qualitative variables were reported as absolute and percentage prevalence and were compared using the χ2 test or Fisher’s exact test, as appropriate.

UW-mRS scores were calculated only for patients alive during follow-up using an ordinary least squares regression model, with mRS scores as discrete ordinal dummy variables and EQ-5D scores as the continuous response variable, adhering to the methodology established by prior studies (16). UW-mRS scores were obtained and validated separately for acute ischemic stroke, intracerebral hemorrhage, and by sex. A simple linear regression analysis was performed to identify variables associated with UW-mRS scores. Multivariable linear regression models were subsequently used to evaluate factors influencing UW-mRS, including both variables significantly correlated in the simple analysis and those considered clinically relevant. This included sex, age over 70, low socioeconomic status, urban residence, prior disability (mRS 3–5), stroke type, and an NIHSS score above 5 at admission. An alfa level of 5% (*p* < 0.05) was considered significant, and 95% confidence intervals were used. Data were processed using STATA software (version 18.5).

## Results

Of the 1,103 patients who experienced a stroke between 2015 and 2016, 890 were a first-ever stroke. At 180 days post the acute event, 773 patients were evaluated, with a 13% loss to follow-up. Baseline characteristics are summarized in Table 1. The cohort consisted of 398 (51%) females, with a mean age of 70.6 years (14.1). Nearly half of the patients (386, 49.9%) had less than 12 years of formal education, and 533 (82%) were classified as having low socioeconomic status based on their public health insurance classification (17). 536 (65%) patients experienced an AIS, had a median NIHSS score of 5 (IQR 3–11), and a median hospital stay of 9 days (IQR 4–15).

**Table 1:** Baseline characteristics of the 773 patients with first ever stroke (FES) followed at 180 days.

Demographics (*n*, %)	
Age, mean SD	70.6 (14.10)
Female	398 (51)
<12 years of formal education	386 (49.93)
Occupation	
Homemaker	166 (21)
Dependent work	63 (8)
Self-employment	97 (13.5)
Pensioner	96 (12.5)
Unknown	347 (45)
Urban resident^a^	248 (32)
Low socioeconomic status^b^	533 (82)
Premorbid modified Rankin Scale (*n*, %)	
0–2	171 (32)
3–5	47 (8)
Unknown	374 (60)
Risk factors (*n*, %)	
Hypertension	498/619 (80)
Atrial fibrillation	58/617 (9)
Diabetes mellitus	221/618 (36)
Acute coronary syndrome	44/616 (7)
Hypercholesterolemia	56/617 (9)
Stroke subtype (*n*, %)	
Acute ischemic stroke	536 (69)
Intracerebral hemorrhage	106 (14)
Subarachnoid hemorrhage	44 (5)
Cerebral venous thrombosis	4 (1)
Undetermined	83 (11)
Stroke severity (median, IQR)	
NIHSS at admission (*n* = 494)	5 (3–11)
Glasgow coma scale (*n* = 274)	15 (14–15)
Median time of hospitalization in days (IQR)	9 (4–15)
Characteristics at 180 days (*n*, %)	
Modified Rankin Scale (mRS)	
0–2	341 (41)
3–5	165 (21)
6	267 (35)
Any change in EQ-5D-3L (level 1 or 2)^c^	
Mobility (*n* = 506)	279 (53)
Self-Care (*n* = 505)	181 (36)
Usual activities (*n* = 504)	257 (53)
Pain/Discomfort (*n* = 502)	336 (67)
Anxiety/Depression (*n* = 499)	263 (53)

^aBased on the sociodemographic characterization of the patient’s municipality of residence at the time of the 2017 census. bAmong those with National Healthcare insurance; NIHSS, National Institutes of Health Stroke Scale; cDead patients or patients with no information were excluded.^

**Figure 1:**
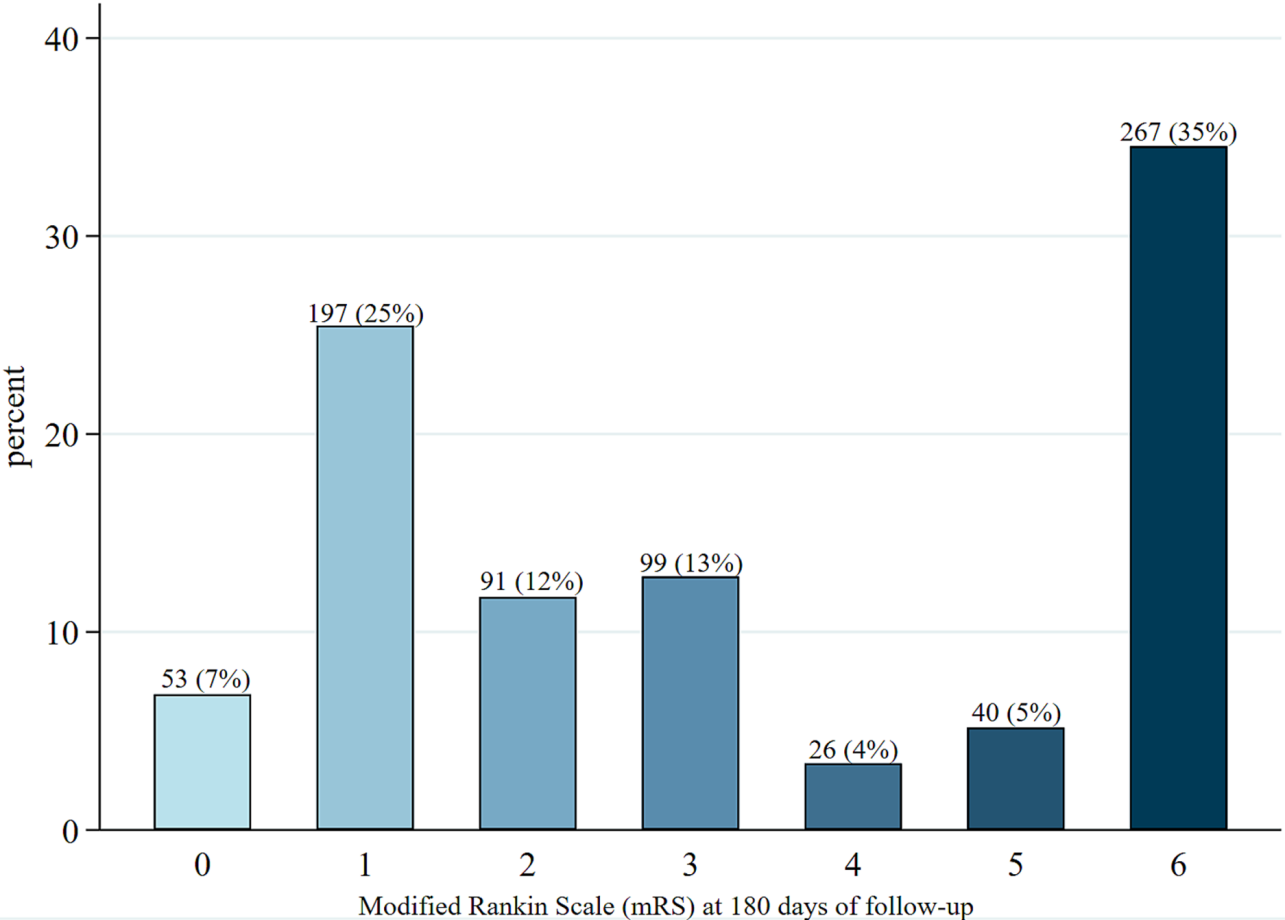
Modified Rankin Scale (mRS) at 180 days after the acute event in the 773 patients.

At 180 days post-acute event, 41% of patients had an mRS score of 0–2, 21% had a score of 3–5, and 35% had died (Table 1; Figure 1). Among patients with hemorrhagic stroke, 62% died, compared to 21% of those with an AIS (*p* < 0.001). No significant differences were observed in the distribution of mRS scores by sex (Supplementary Figures 1, 2).

**Figure 2:**
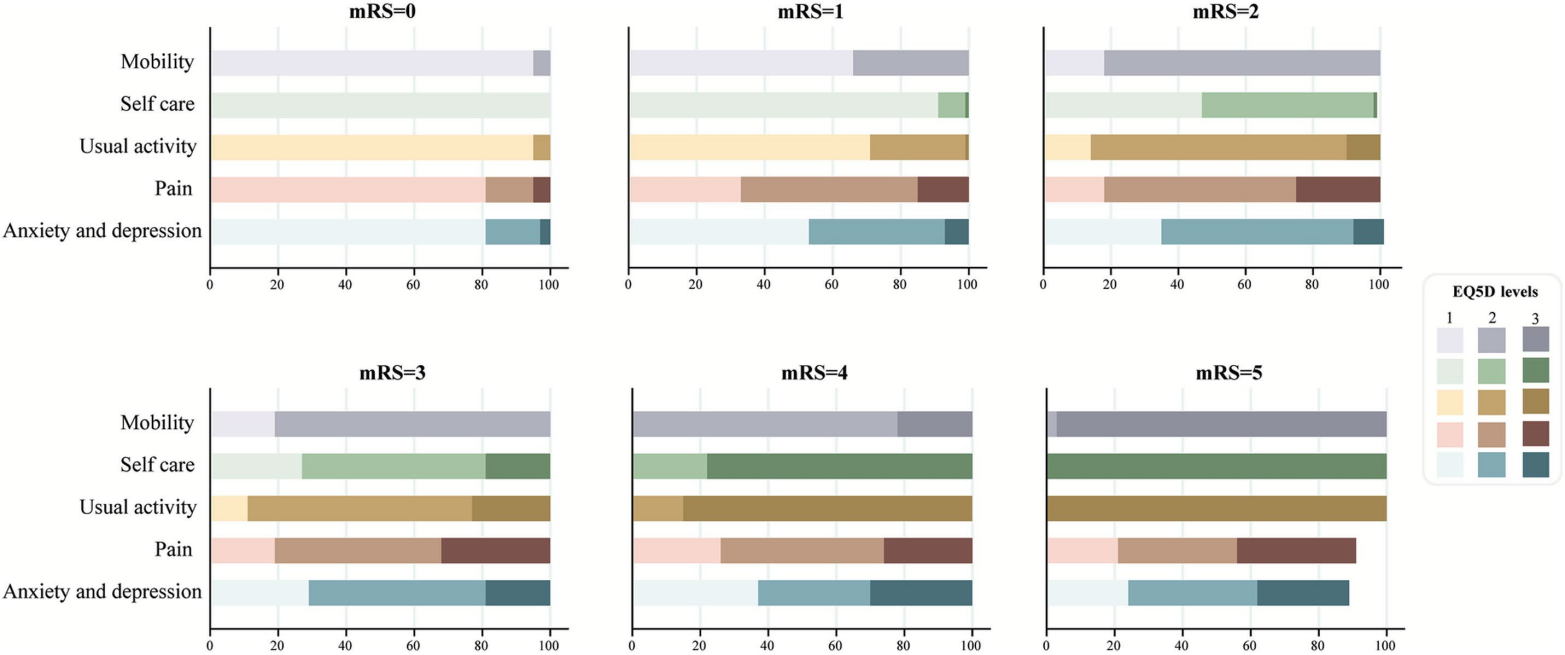
European quality of life 5-dimensional questionnaire utility scores by modified Rankin Scale scores at 180 days of follow up.

In the EQ-5D-3L assessment, the most affected dimension was pain/discomfort (67%), followed by mobility and anxiety/depression (53%). Figure 2 shows the distribution of the EQ-5D-3L for each mRS category. There was a strong negative association between mRS and EQ-5D-3L index values overall (*r* = −0.82; *p* < 0.001; Supplementary Figure 3).

**Figure 3:**
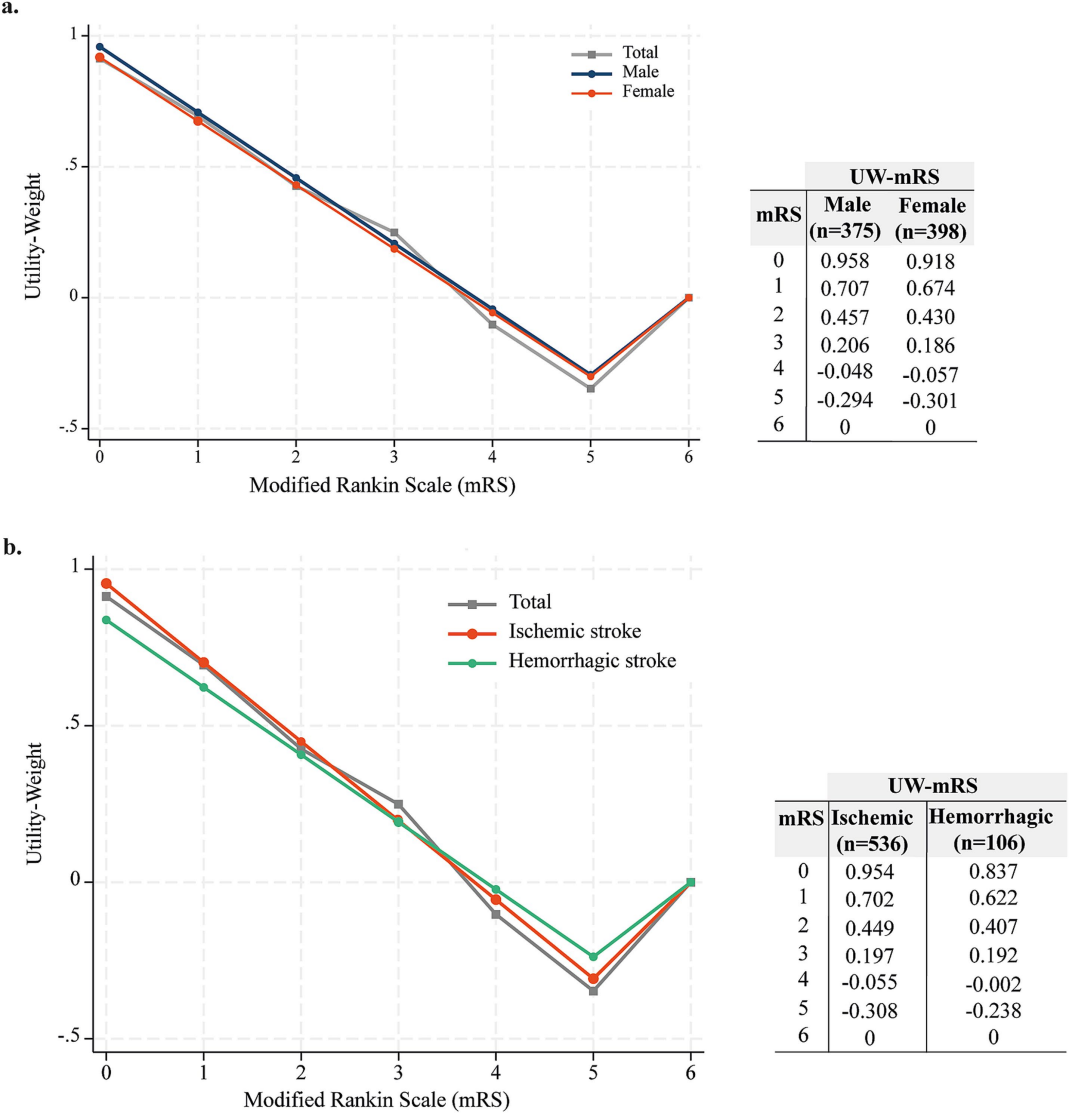
UW-mRS score derived from the regression model by sex **(a)**, and stroke subtype **(b)**.

The UW-mRS values, calculated from the mean EQ-5D-3L utility scores from the Chilean population (15), across mRS categories 0–6 at 180 days, were: 0.913, 0.694, 0.425, 0.249, −0.102, −0.347 and 0, respectively (Table 2). When disaggregated by sex, females tended to have slightly lower UW-mRS values compared to males, though this difference was not statistically significant (*p* = 0.194, Figure 3a). In terms of stroke type, ischemic stroke survivors had lower UW-mRS scores than those with hemorrhagic stroke at 180 days post-acute event (Figure 3b).

**Table 2:** UW-mRS values derived for each category of the modified Rankin scale.

mRS	UW-mRS	SD
0	0.913	0.157
1	0.694	0.234
2	0.425	0.213
3	0.249	0.294
4	−0.102	0.267
5	−0.347	0.127
6	0	0

mRS, modified Rankin Scale; UW-mRS, utility-weighted modified Rankin Scale; SD, standard deviation.

**Figure 4:**
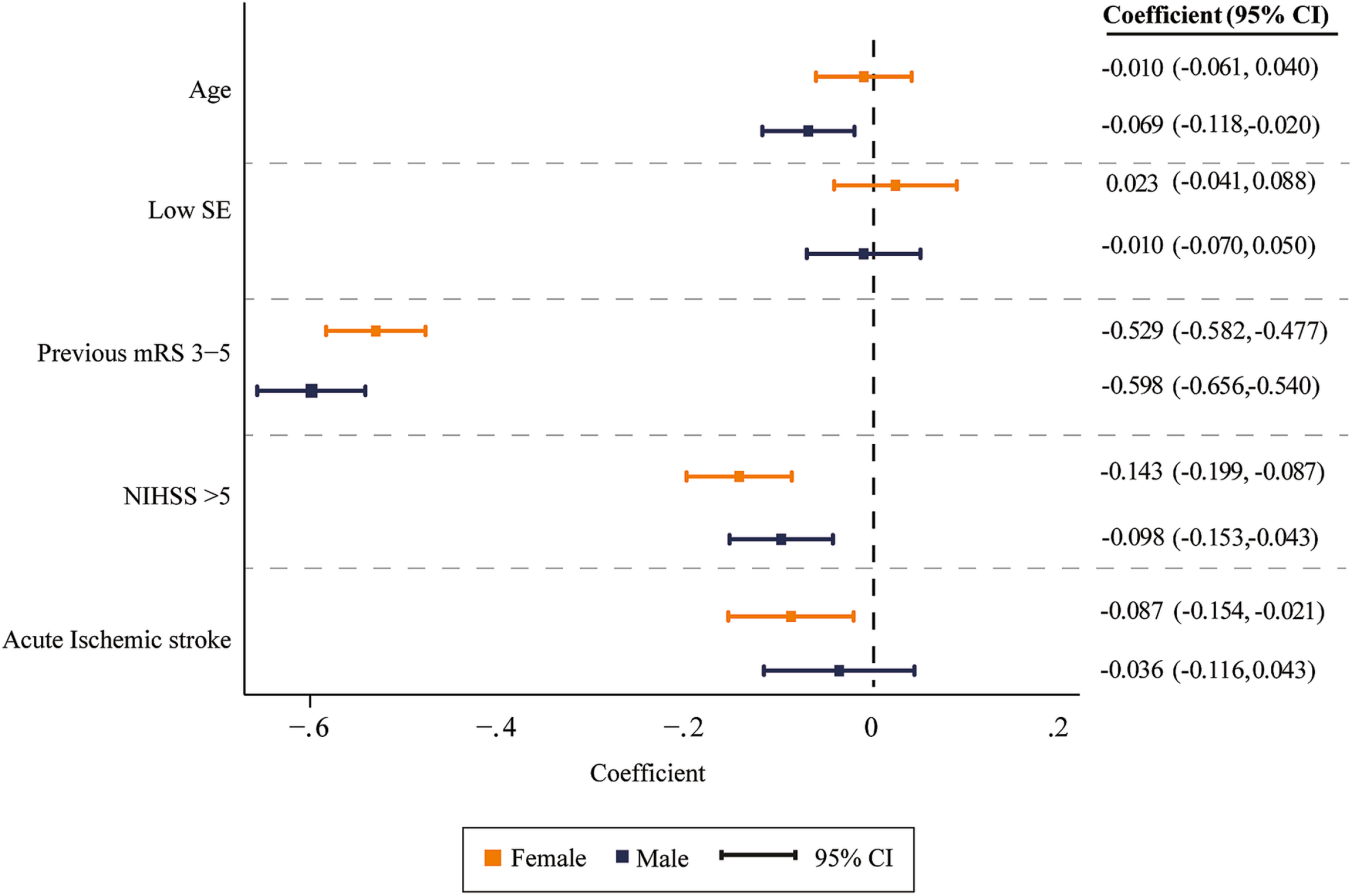
Multivariable model coefficients assessing risk factors by sex with 95% confidence intervals.

A linear regression analysis was conducted to explore the relationship between UW-mRS scores and key variables. In the simple regression, significant associations were found between age > 70 years (Coefficient *β* [β] −0.007 [Standard error SE] 0.001, *p* < 0.001), lower socioeconomic status (β −0.075 [SE 0.040], *p* < 0.001), previous mRS score of 3–5 (β −0.607 [SE 0.018], *p* < 0.001), ischemic stroke subtype (*β* −0.025 [SE 0.041], *p* < 0.001), and NIHSS >5 at admission (*β* −0.273 [SE 0.028], *p* < 0.001) with worse outcome (Table 3). In the multivariable model, age > 70 years (β −0.038 [SE 0.018], *p* = 0.032), previous mRS score of 3–5 (β −0.556 [SE 0.197], *p* < 0.001), ischemic stroke (β −0.066 [SE 0.025] *p* = 0.010), and NIHSS >5 at admission (β −0.015 [SE 0.002], *p* < 0.001) remained significant predictors of lower UW-mRS scores, with an R^2^ of 70%. When the multivariable model was disaggregated by sex to assess potential differences, the previous mRS score of 3–5 and NIHSS >5 at admission were associated to worse UW-mRS in both sexes (Supplementary Table 1). Distinctly, age > 70 years was significant for males (β −0.069 [SE 0.024], *p* = 0.006) and having an AIS was significant for females (β −0.087 [SE 0.033], *p* = 0.010) (Figure 4; Supplementary Table 1). The model explained a similar proportion of variance in both groups, with an R^2^ of 69% for females and 72% for males (Supplementary Table 1).

**Table 3:** Results of simple and multivariable linear regression models analyzing factors associated with UW-mRS scores.

	Simple			Multivariable model		
	Coefficient β	Standard error	*p* value	Coefficient β	Standard error	*P* value
Sex	0.059	0.030	0.052	−0.006	0.171	0.710
Age > 70 years old	**−0.007**	**0.001**	**<0.001**	**−0.038**	**0.018**	**0.032**
<12 years of formal education	−0.029	0.030	0.347	−0.010	0.017	0.546
Low socioeconomic status^a^	**−0.075**	**0.040**	**<0.001**	0.006	0.022	0.781
Urban resident	−0.007	0.032	0.812	0.009	0.017	0.613
Previous mRS 3–5	**−0.607**	**0.018**	**<0.001**	**−0.556**	**0.197**	**<0.001**
Acute ischemic stroke	**−0.025**	**0.041**	**<0.001**	**−0.066**	**0.025**	**0.010**
NIHSS at admission >5	**−0.273**	**0.028**	**<0.001**	**−0.015**	**0.002**	**<0.001**
R^2^: 70%						

^aAmong those with National Healthcare insurance; NIHSS, National Institutes of Health Stroke Scale.^

Bold values indicate statistically significant *p*-values (*p* < 0.05).

## Discussion

The present study examined the distribution of health outcomes in a Chilean population-based cohort of patients who suffered an acute stroke. To our knowledge, this study is the first to derive a quality-of-life scale, like the EQ-5D-3L, using the UW-mRS in a community-based study, incorporating the patient perspective outside of controlled clinical settings. At 180 days post-stroke, 35% of patients had died, and among the survivors, the most affected dimension of the EQ-5D-3L were pain/discomfort, followed by mobility and anxiety/depression. These results align with studies comparing healthy populations in other countries within the region (18) and in countries like China (19).

The UW-mRS values demonstrated a gradual decline in utility as mRS scores increased, reflecting the expected deterioration in health-related quality of life as disability worsened. These findings corroborate those of Wang et al., who applied similar methodologies based on cohorts from clinical trials (16). Their reported utility values for mRS scores 0–6 were 0.96, 0.88, 0.74, 0.56, 0.25, −0.11, and 0, respectively. Notably, the utility values for mRS 4 and 5 were significantly lower in the Chilean population, resulting in negative values, which suggest a more severe perception of quality of life at the same mRS level compared to Wang et al.’s cohort. This variation may be attributed to cultural differences in health perception, disparities in access to healthcare, or other socioeconomic factors (7, 14) as well as the methodological differences between studies that derived the EQ-5D-3L value sets (18, 20). These findings underscore the importance of using population-specific utility values when calculating UW-mRS scores, as the choice of value set can significantly influence results and their interpretation, with important implications for clinical practice and research.

When analyzing the UW-mRS scores by sex, females were found to report worse health status than males for the same level of motor disability, though the differences were not statistically significant. Previous studies indicate that, on average, females score 0.03 points lower than males (21). These discrepancies may be explained by the influence of distinct cultural and social factors that shape how females perceive and report their health status (22), as well as to higher levels of anxiety or depression, pain, and discomfort compared to males (23). Interestingly, age over 70 years emerged as a significant predictor of worse UW-mRS scores in males, which may be explained by the fact that, at the time of stroke, females were significantly older than males (mean age 72.17 vs. 68.94 years respectively). Additionally, ischemic stroke was a significant predictor of poorer outcomes in females. Although the proportion of ischemic stroke was similar between sexes, a higher percentage of females who suffered ischemic stroke (30%) had mRS scores between 3 and 5 compared to males (21%), and the higher UW-mRS weights for ischemic stroke may have further accentuated its impact on females.

When comparing UW-mRS scores by stroke type, we found that the values for ischemic stroke were lower than those for ICH, and this impact was more relevant in women. The difference may be attributed to the greater severity typically associated with ICH and the higher early mortality rate among ICH patients during follow-up.

This study has strengths and limitations that must be acknowledged. Among its strengths, we identified key predictors of UW-mRS scores in stroke survivors, including age, prior mRS score, and NIHSS at admission, which aligns with previous findings (24). Notably, age over 70 years emerged as a significant predictor only in males, while acute ischemic stroke had a greater impact on females. These sex-specific insights are crucial for tailoring personalized treatment and rehabilitation strategies. The use of a population-based cohort from a low-income setting adds to the relevance of the findings, providing valuable reference data for understanding stroke recovery in real-world conditions and informing future healthcare policies.

However, there are also important limitations. The data come from a single population-based cohort in Chile, which may limit the generalizability of the findings. Despite this, the results are representative of a low-income population with high stroke risk factors and could serve as a reference for future population-based studies. The follow-up was conducted by telephone, though studies have validated this method’s effectiveness (25), and it was carried out by trained personnel. Additionally, we lacked consistent information on access to rehabilitation or post-stroke care, which may have influenced the reported quality of life perceptions. Lastly, using the ordinary least squares regression model to derive UW-mRS scores may not fully capture the complexity of outcomes across stroke subtypes and demographics. Future research should explore alternative models and validate the UW-mRS in diverse populations.

## Conclusion

These results present UW-mRS values derived from a population-based stroke study, further supporting UW-mRS as a reliable measure of PCOs in post-stroke patients. Key determinants of health-related quality of life included age, prior disability, and stroke severity, with age over 70 years being a significant predictor for males and AIS having a greater impact on females. Incorporating UW-mRS as a PCO in future stroke research and clinical practice may provide a more nuanced understanding of the impact of stroke on survivors, offering valuable insights for clinical decision-making and rehabilitation strategies across diverse healthcare settings.

## Data Availability

The raw data supporting the conclusions of this article will be made available by the authors, without undue reservation.
