# Supplementary material for: Patient centered outcomes in stroke: utility-weighted modified Rankin Scale results in a community-based study
Source: Front Neurol. 2025 Mar 21;16:1539107. doi: 10.3389/fneur.2025.1539107 (PMC11968358; doi:10.3389/fneur.2025.1539107)
Supplement: Supplementary file 1 [file Supplementary_file_1.docx]

Supplementary Material

**Stroke Outcomes using Utility-Weighted Modified Rankin Scale Scores: Results from a large community-based study in Chile**

Carlos Delfino, Paula Muñoz Venturelli, Gabriel Cavada, Lorena Hoffmeister, Pablo Lavados

- Supplementary figures: 3
- Supplementary tables: 1

# **Supplementary Figure 1**. 180-Day modified Rankin scale (mRS) scores across different stroke types


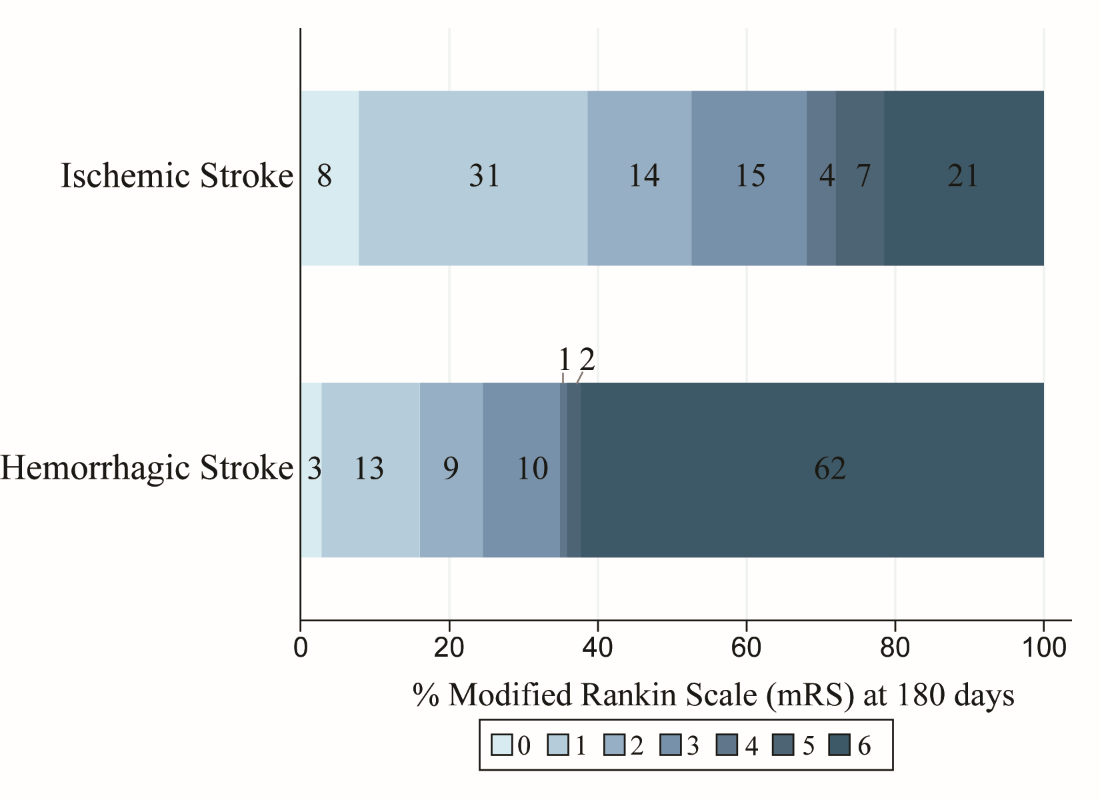


p <0.001

**Supplementary Figure 2**. Sex-specific percentage distribution of modified Rankin Scale (mRS) at 180 days of follow-up, stratified by stroke type.


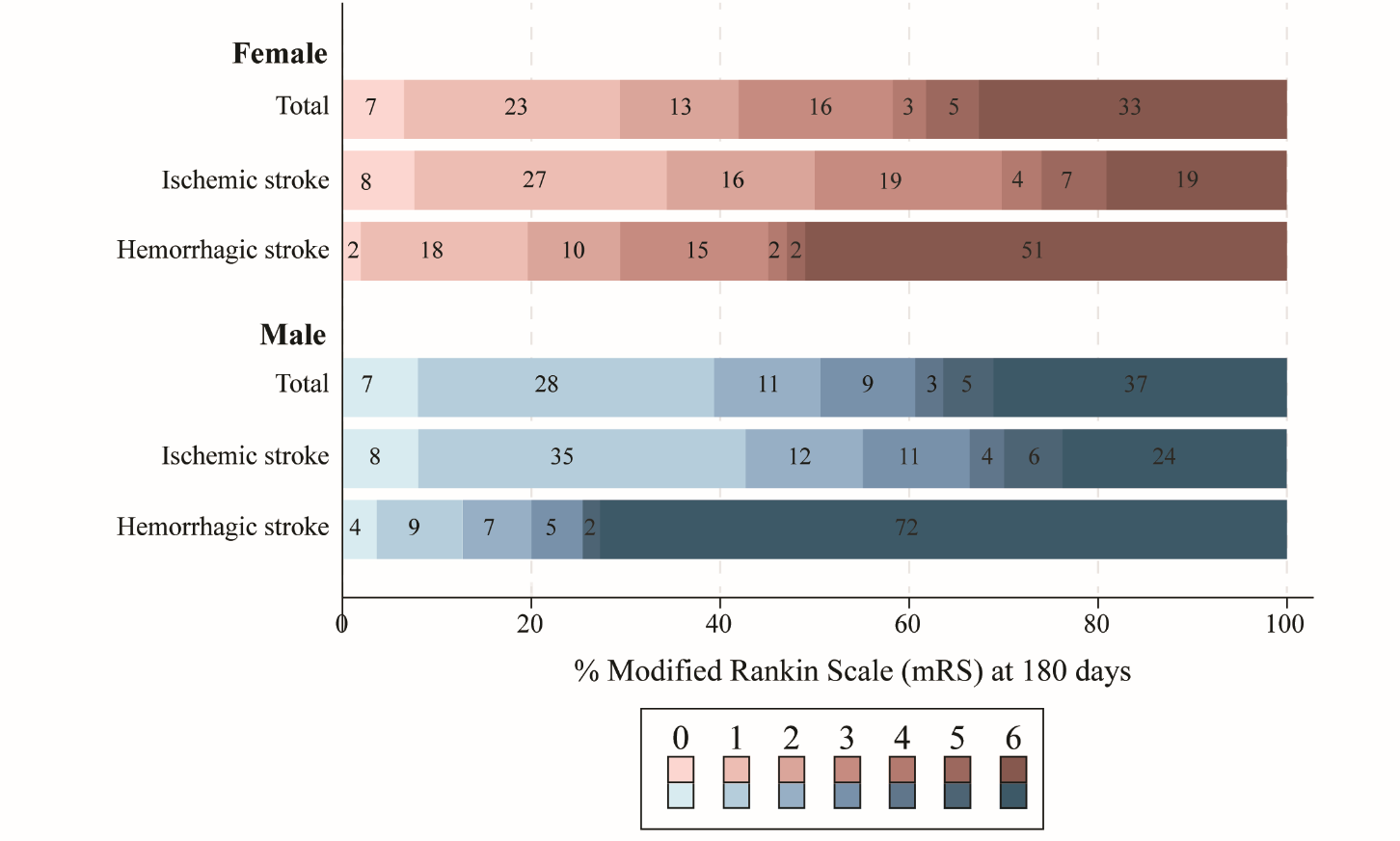


p=0.066

# **Supplementary Figure 3.** Distribution of EQ-5D-3L utility index values according to modified Rankin scale (mRS) at 180 days

note: Values of mRS 6 were arbitrarily assigned to a zero index value.

# **Supplementary table 1.** Multivariable linear regression models in females and males to analyse factors associated with UW-mRS scores.

|  | **Females** | | | **Males** | | |
| --- | --- | --- | --- | --- | --- | --- |
|  | **Coefficient** | **Standard error** | **P value** | **Coefficient** | **Standard error** | **P value** |
| Age >70 years old | -0.010 | 0.025 | 0.691 | **-0.069** | **0.024** | **0.006** |
| Low socioeconomic status^a^ | 0.023 | 0.033 | 0.482 | -0.010 | 0.030 | 0.734 |
| Previous mRS 3-5 | **-0.529** | **0.026** | **< 0.001** | **-0.598** | **0.029** | **< 0.001** |
| Acute ischemic stroke | **-0.087** | **0.033** | **0.010** | -0.036 | 0.040 | 0.370 |
| NIHSS at admission >5 | **-0.143** | **0.028** | **< 0.001** | **-0.098** | **0.027** | **0.001** |
| R^2^ | **69%** | | | **72%** | | |
| **^a^** Among those with National Healthcare insurance; NIHSS, National Institutes of Health Stroke Scale. | | | | | | |
